# Family coping profiles in patients with chronic heart failure: a latent profile analysis

**DOI:** 10.3389/fpsyg.2026.1882861

**Published:** 2026-07-27

**Authors:** Xiong Zhang, Yimei Zhang, Xu Su, Wei Wei, Yangjuan Bai, Fang Ma

**Affiliations:** 1 Department of Geriatric General Surgery, The First Affiliated Hospital of Kunming Medical University, Kunming, Yunnan, China; 2 Department of Critical Care Medicine, The First Affiliated Hospital of Kunming Medical University, Kunming, Yunnan, China; 3 Department of Psychiatry, The First Affiliated Hospital of Kunming Medical University, Kunming, Yunnan, China; 4 Department of Digestive Surgery, The First Affiliated Hospital of Kunming Medical University, Kunming, Yunnan, China; 5 Department of Cardiology, The First Affiliated Hospital of Kunming Medical University, Kunming, Yunnan, China; 6 Department of Nursing, The First Affiliated Hospital of Kunming Medical University, Kunming, Yunnan, China

**Keywords:** family coping, psychological, chronic heart failure, latent profile analysis, nursing

## Abstract

**Background:**

Family coping is integral to chronic heart failure management, yet systematic research on how families collectively cope remains limited. This study aimed to identify distinct family coping patterns among CHF patients and examine their associations with sociodemographic factors, fatigue, and family functioning.

**Design:**

This cross-sectional study was conducted in China.

**Methods:**

Patients with chronic heart failure were recruited through convenience sampling from tertiary hospitals in Yunnan Province, southwestern China. Participants completed questionnaires assessing sociodemographic characteristics, family coping (FCS-CHF), fatigue (CChFS), and family functioning (CGFFS). Latent profile analysis was conducted to identify distinct coping subgroups, followed by chi-square tests, t-tests, and binary logistic regression to examine the factors associated with the profile membership of these subgroups.

**Results:**

A total of 536 patients were included in this study. Latent profile analysis identified two distinct coping patterns among families: constructive family coping (56.7%) and depleting family coping (43.3%). The constructive family coping pattern consistently scored higher across all six coping dimensions. Binary logistic regression revealed that family structure, family size, annual household income, and family functioning were significantly associated with the type of coping pattern adopted by families. Specifically, better family functioning (OR = 0.311, *p* < 0.001), a medium family size (3–6 persons; OR = 0.427, *p* = 0.004), and higher income (≥200,000 CNY; OR = 0.415, *p* = 0.025) were associated with a lower likelihood of depleting family coping. In contrast, a nuclear family structure was associated with a higher likelihood of depleting family coping (OR = 1.739, *p* = 0.048).

**Conclusion:**

This study provides empirical evidence of two distinct family coping profiles among patients with chronic heart failure in a rural resource-limited population. The findings highlight the need for tailored, family centered nursing interventions that address specific support needs, including comprehensive multicomponent support for depleting families and reinforcement of existing strengths for constructive families. Future research should extend these findings through multi-informant designs that capture both patient and family member perspectives and through replication in more diverse populations to establish their generalizability to other populations.

## Background

Chronic heart failure (CHF) is a progressive and debilitating condition that imposes profound limitations on patients’ physical capacity, including dyspnea, fatigue, and edema, while also elevating the risk of anxiety and depression, which collectively erode the quality of life (Cook et al., 2014; Dramba et al., 2025; Polikandrioti and Tsami, 2025). Globally, CHF affects an estimated 1%–3% of the adult population, with a prevalence exceeding 56 million cases worldwide, a number that continues to rise (Jia and Du, 2025; Zhang and Feng, 2025). The impact on families is equally severe. Family caregivers face intense daily caregiving demands, from medication management to emotional support and symptom monitoring, alongside persistent psychological strain and an accumulating financial burden (Grant and Graven, 2018; Choi et al., 2021; Suksatan et al., 2022). Over time, this sustained caregiving load compromises caregivers’ physical and mental health, disrupts family functioning, and undermines overall household well-being (Kim et al., 2020; Reinhard and Brassard, 2020).

Coping, defined as cognitive and behavioral efforts to manage stressors that exceed personal resources (Folkman, 2013), can be meaningfully extended to the family level. Family coping refers to the coordinated processes through which a family system collectively navigates major stressors, maintains functional integrity, and supports members’ well-being (McCubbin and McCubbin, 1989; Patterson, 2002; Alianiello, 2005). These processes are uniquely demanding for families managing CHF. Families of patients with CHF require simultaneous attention to complex treatment regimens and emotional regulation to preserve homeostasis (Mårtensson et al., 2001; Hu et al., 2023). Consequently, effective family coping is not merely beneficial but essential for CHF management; when coping falters, patient outcomes worsen, caregiver burnout escalates, and a self-reinforcing cycle of deteriorating disease management and family distress may take hold (Ma, 2019).

Based on the trichotomy of coping strategies proposed by Weiten and our team’s preliminary research, family coping of patients with CHF comprises management strategies, psychological coping, family members’ support, emergency coping, heart failure awareness, and patients’ health behavior (Ma, 2019; Zhang et al., 2026). Family coping in patients with CHF may exhibit significant differences in the specific manifestations of diverse components of family coping. It has been evidenced that family coping profiles of patients with CHF are distinct and characterized by heterogeneity, highlighting the critical need to understand these heterogeneous coping patterns to develop tailored interventions that address specific needs and ultimately improve patient and family outcomes.

Identifying distinct family coping profiles is essential for designing targeted family-centered interventions. Latent profile analysis (LPA) is a person-centered method that identifies subgroups based on response patterns across multiple indicators (Gorbachevsky et al., 2025). Unlike variable-centered approaches, which assume population homogeneity, LPA can reveal heterogeneous coping patterns that inform tailored support strategies (Spurk et al., 2020). Clinical reality suggests that families confronting objectively similar disease demands often exhibit markedly different coping configurations—some excel in psychological coping but lack emergency preparedness, while others mobilize strong family support yet struggle with health behavior management (Zhang X. et al., 2024; Zhang et al., 2026). LPA preserves the multidimensional integrity of each family‘s coping profile without imposing arbitrary cut-points, making it uniquely suited to answer whether distinct family coping configurations exist among patients with CHF and whether these configurations are differentially associated with family functioning and fatigue. Among the factors potentially shaping these profiles, high-quality family functioning appears to serve as a protective resource (Zhang X. et al., 2024), whereas fatigue—a hallmark symptom of CHF—undermines patients’ capacity for self-management and contributes to caregiver exhaustion, thereby eroding the family‘s collective coping reserves (Pavlovic et al., 2022; Uğurlu et al., 2024). Although LPA has been applied in CHF research to delineate patient subgroups based on symptoms, treatment adherence, and quality of life (Chen et al., 2024; Ren et al., 2024; Yi et al., 2025), its use in examining family coping profiles remains unexplored. Accordingly, this study employed LPA to identify distinct family coping profiles and examine the sociodemographic and clinical factors associated with profile membership.

## Methods

### Aims

This study used LPA to identify different categories of family coping among patients with CHF and examined the influence of demographic characteristics, fatigue, and family functioning.

### Design

A cross-sectional design was used in accordance with the Strengthening the Reporting of Observational Studies Guidelines.

### Participants

Participants were recruited through convenience sampling from multiple tertiary hospitals in Yunnan Province, southwestern China. The eligibility criteria were as follows: (1) age ≥ 18 years; (2) meeting the diagnostic criteria for heart failure specified in the 2022 American Heart Association/American College of Cardiology/Heart Failure Society of America guidelines (Heidenreich et al., 2022); (3) classified as New York Heart Association (NYHA) functional class I–III; (4) demonstrating adequate comprehension and verbal communication skills in Chinese; and (5) providing written informed consent. The exclusion criteria were as follows: (1) acute cardiovascular events (e.g., myocardial infarction, pulmonary embolism, or systemic infection) within the preceding month; (2) severe comorbid conditions, such as malignant tumors or renal failure; and (3) documented cognitive impairment or psychiatric disorders.

Patients with NYHA class IV were excluded for reasons of clinical stability and ethical considerations. These individuals, who are symptomatic even at rest, frequently experience severe dyspnea, fatigue, and hemodynamic instability (Gorbachevsky et al., 2025), which would make the completion of a 20–30 min questionnaire unduly burdensome. All criteria were applied to safeguard the participants’ welfare and ensure data quality. The required sample size for LPA is generally considered to be between 300 and 500 individuals (Ferguson et al., 2020).

### Data collection

From October 2023 to February 2024, convenience sampling was used to identify patients with CHF from 11 tertiary-level Grade A hospitals in Southwest China. A total of 560 participants were recruited for this study. Of these, 550 were eligible to participate and complete the survey, and 14 were excluded because of significant inconsistencies in their answers to the logically related items.

The survey instrument consisted of two sections: the first section collected sociodemographic characteristics of the participants. Subsequent sections assessed the following constructs: family coping was evaluated using the Family Coping Scale for Patients with Chronic Heart Failure (FCS-CHF), fatigue levels were measured using the Chinese version of the Chalder Fatigue Scale (CChFS), and family functioning was appraised using the Chinese version of the General Family Functioning Scale (CGFFS).

Data were collected by trained research staff in hospital wards in strict adherence to the predefined eligibility criteria. To ensure consistency and minimize interviewer bias, all staff members received standardized training on administering the survey without influencing the participants’ responses.

A mixed-mode strategy was adopted for data collection. Although most participants completed paper-based questionnaires, some responses were collected via the REDCap electronic platform, which enabled real-time monitoring of data completeness. Participants who could complete the survey independently did so, while those who required assistance owing to their educational level, health status, or personal preference had the questionnaire items read aloud to them by trained staff who recorded their answers accordingly.

To enhance data quality, each questionnaire was checked for completeness immediately after it was completed. Participants were asked to respond to any items that were inadvertently skipped on-site. A double-entry procedure was employed for data entry: two researchers independently entered the data, and the resulting datasets were compared to identify and correct any discrepancies.

### Measures

#### Sociodemographic characteristics questionnaire

The sociodemographic characteristics selected for the study were chosen based on a review of the literature and their relevance to the study’s aims. The following information was collected using a self-designed questionnaire developed by a multidisciplinary team (comprising nursing, psychology, management, and public health specialists): sex, age, marital status, place of residence, living arrangements, educational level, family structure, and annual household income.

#### Family coping scale for patients with chronic heart failure (FCS-CHF)

Family coping was assessed using the FCS-CHF, a 24-item instrument with item 15 as reverse-scored developed and validated to measure family coping in the context of CHF management (Zhang X. et al., 2024; Zhang et al., 2026). Although the scale is designed for administration to both patients and family members, the present study focused specifically on the patient’s perspective. The FCS-CHF comprises six dimensions: strategies for better CHF management (six items), psychological coping (five items), substantial support from family members (four items), emergency coping (three items), overall heart failure awareness (three items), and patients’ health behaviors (three items). Items are rated on a 7-point Likert scale (1 = very inconsistent, 7 = very consistent), with 15 reverse-scored items; higher total scores (range: 24–168) indicate more adaptive perceived family coping. The scale has demonstrated robust psychometric properties, including good structural validity and internal consistency. The Cronbach’s *α* coefficient for the FCS-CHF was 0.902, and the split-half reliability coefficient was 0.846. Exploratory factor analysis (EFA) yielded a Kaiser-Meyer-Olkin (KMO) measure of 0.909, with Bartlett’s test of sphericity resulting in χ^2^ = 4591.112 (df = 276, *p* < 0.001). The cumulative explained variance was 67.108%. Confirmatory factor analysis (CFA) showed a χ^2^/df ratio of 2.707 and a root mean square error of approximation (RMSEA) of 0.071. In the present sample, Cronbach’s *α* was 0.896, indicating satisfactory reliability (Zhang X. et al., 2024).

Although FCS-CHF is designed for administration to both patients and family members, the present study focused specifically on the patient‘s perspective. This focus is clinically meaningful, as patients’ perceived coping resources are closely linked to their psychological and behavioral outcomes. From a family systems perspective, however, family coping is ideally assessed using multi-respondent data (e.g., patient–family member dyads). Practical constraints, including family member unavailability, precluded multi-respondent data collection in this study. Future research should therefore adopt dyadic designs to capture both patient and family member perspectives, enabling analysis of perceptual congruence and discrepancy.

#### The Chinese version of the Chalder Fatigue Scale (CChFS)

The CChFS was developed by Chalder et al. (1993) in the UK and was used to assess the severity of fatigue. This version has been verified in China, where it has demonstrated satisfactory reliability and validity (Wong and Fielding, 2010). The scale has 11 items, and the answer options for each item are “yes” or “no” which are scored as 1 point and 0 points, respectively. Question 11 was scored in reverse. The maximum total score was 11 points. Higher scores indicate more severe fatigue. The instrument had good internal consistency (Cronbach’s *α* = 0.863). The Cronbach’s alpha coefficient for the CChFS in this study was 0.75.

#### The Chinese version of General Family Functioning Scale (CGFFS)

The CGFFS is a subscale of the McMaster Family Assessment Device (FAD). It was translated and adapted by Yuan and Zhang (2019) and validated for use in China. Its applicability to the Chinese population was analyzed; however, a reliability analysis was not performed. Nonetheless, confirmatory factor analysis (CFA) indicated a favorable model fit, as reflected by a comparative fit index (CFI) of 0.923, Tucker–Lewis index (TLI) of 0.922, and root mean square error of approximation (RMSEA) of 0.068. The scale comprises 12 items on a four-point self-report scale. The mean score for each item constituted the total scale score, with higher scores indicating better functioning. In this study, the Cronbach’s alpha coefficient for the CGFFS was 0.75.

### Data analysis

To identify distinct family coping profiles, a series of latent profile analyses (LPAs) was conducted using Mplus 8.3, with the six FCS-CHF dimensions as indicators. The models were estimated sequentially, starting with a one-profile solution and increasing the number of profiles until the optimal solution was reached. The optimal model was selected based on multiple fit indices following established recommendations (Tein et al., 2013): (1) lower values of the Akaike information criterion (AIC), Bayesian information criterion (BIC), and adjusted BIC (aBIC) indicated better relative fit; (2) the Lo–Mendell–Rubin (LMR) test and bootstrap likelihood ratio test (BLRT) compared the fit of a k-profile model against a k-1-profile model, with a significant *p*-value favoring the more complex model; and (3) entropy ≥ 0.80 indicated good classification accuracy.

Once the optimal profile solution was determined, the Bolck–Croon–Hagenaars (BCH) method was used to examine the associations between profile membership and distal outcomes, while accounting for classification uncertainty (Bolck et al., 2004; Asparouhov and Muthén, 2014). Subsequently, binary logistic regression was performed to identify the sociodemographic factors associated with profile membership. All subsequent analyses were performed using SPSS (version 27.0). Demographic characteristics were summarized using frequencies and percentages, and continuous variables (CChFS and CGFFS scores) were described using means and standard deviations. Chi-square tests were used to compare demographic differences between profiles. Variables showing significant associations (*p* < 0.05) in univariate comparisons were entered as independent variables into a binary logistic regression model, with latent profile category as the dependent variable, to identify factors associated with membership in specific family coping profiles.

## Results

### Participants’ characteristics

A total of 550 participants completed the survey. After rigorous data screening, 14 questionnaires were excluded due to significant inconsistencies in logically related items, yielding a final sample of 536 (response rate: 97.45). The mean age was 67.83 years (SD = 12.95; range: 21–97). The sample included 311 men (58.02%) and 225 women (41.98%) participants. Additional sociodemographic characteristics are presented in Table 1.

**Table 1:** The descriptive statistics for the full sample and each latent profile.

Variables	Categories	Total (*N* = 536)	Constructive family coping (*N* = 304)	Depleting family coping (*N* = 232)	χ^2^	*p*
Sex	Male	311 (58.02)	184 (60.53)	127 (54.74)	1.808	0.179
	Female	225 (41.98)	120 (39.47)	105 (45.26)		
Ethnicity	Han	447 (83.40)	261 (85.86)	186 (80.17)	3.069	0.080
	Minority	89 (16.60)	43 (14.14)	46 (19.83)		
Age	<65 years	201 (37.50)	117 (38.49)	84 (36.21)	0.292	0.589
	≥65 years	335 (62.5)	187 (61.51)	148 (63.68)		
Place of residence	Rural	249 (46.46)	127 (41.78)	122 (52.59)	6.181	0.013
	Urban	287 (53.54)	177 (58.22)	110 (47.41)		
Living arrangement	Living with spouse only	174 (32.46)	94 (30.92)	80 (34.48)	4.496	0.213
	Living with children/multiple generations only	148 (27.61)	78 (25.66)	70 (30.17)		
	Living with spouse and children/multiple generations	149 (27.80)	95 (31.25)	54 (23.28)		
	Other	65 (12.13)	37 (12.17)	28 (12.07)		
Educational level	Primary school or below	286 (53.36)	147 (48.36)	139 (59.91)	7.064	0.008
	Junior high school or above	250 (46.64)	157 (51.64)	93 (40.09)		
Marital status	Married	393 (73.32)	221 (72.70)	172 (74.14)	5.382	0.068
	Widowed	119 (22.20)	64 (21.05)	55 (23.71)		
	Other	24 (4.48)	19 (6.25)	5 (2.16)		
Number of children	≤2	330 (61.57)	184 (60.53)	146 (62.93)	0.322	0.571
	≥3	206 (38.43)	120 (39.47)	86 (37.07)		
Occupation	Farmer or Worker	275 (51.31)	142 (46.71)	133 (57.33)	7.823	0.020
	Retired	179 (33.40)	106 (34.87)	73 (31.47)		
	Other	82 (15.30)	56 (18.42)	26 (11.21)		
Payment method	Employee basic medical insurance	145 (27.05)	87 (28.62)	58 (25.00)	0.879	0.644
	Urban and rural resident basic medical insurance	341 (63.62)	189 (62.17)	152 (65.52)		
	Other	52 (9.70)	28 (9.21)	22 (9.48)		
Self-rated health status	Very good	42 (7.84)	27 (8.88)	15 (6.47)	27.630	<0.001
	Good	143 (26.68)	105 (34.54)	38 (16.38)		
	Fair	299 (55.78)	142 (46.71)	157 (67.67)		
	Poor	52 (9.70)	30 (9.87)	22 (9.48)		
Family model	Nuclear family	190 (35.45)	100 (32.89)	90 (38.79)	8.222	0.042
	Stem family	201 (37.50)	110 (36.18)	91 (39.22)		
	Extended family	101 (18.84)	70 (23.03)	31 (13.36)		
	Other	44 (8.21)	24 (7.89)	20 (8.62)		
Family size	≤2 persons	160 (29.85)	71 (23.36)	89 (38.36)	14.517	<0.001
	3–6 persons	331 (61.75)	207 (68.09)	124 (53.45)		
	≥7 persons	45 (8.40)	26 (8.55)	19 (8.19)		
Annual household income	≤50,000 CNY	210 (39.18)	95 (31.25)	115 (49.57)	29.197	<0.001
	50,001–100,000 CNY	130 (24.25)	73 (24.01)	57 (24.57)		
	100,001–150,000 CNY	71 (13.25)	42 (13.82)	29 (12.50)		
	150,001–200,000 CNY	58 (10.82)	42 (13.82)	16 (6.90)		
	≥210,000 CNY	67 (12.50)	52 (17.11)	15 (6.47)		

### Latent profile analysis

Latent profile models with one to four profiles were estimated and compared (Table 2). Model selection was guided by multiple fit indices, including information criteria (Akaike information criterion [AIC], Bayesian information criterion [BIC], and adjusted BIC [aBIC]), likelihood ratio tests (LMR and BLRT), entropy, and class size (>5% of the sample for stability), as well as interpretability and downstream analytic feasibility.

**Table 2:** Fit statistics for the latent profile analysis.

Profile	AIC	BIC	aBIC	*p*		Entropy	Class probability
				LMR	BLRT		
1 Class	18944.95	18996.36	18958.27	—	—	—	1.000
2 Class	18203.27	18284.67	18224.36	0.0002	<0.0001	0.868	0.433\0.567
3 Class	17904.37	18015.76	17933.22	0.0262	<0.0001	0.923	0.016\0.549\0.433
4 Class	17798.34	17939.72	17834.96	0.0658	<0.0001	0.885	0.017\0.160\0.271\0.552
5 Class	17718.16	17889.53	17762.56	0.3217	<0.0001	0.911	0.017\0.017\0.280\0.549\0.138

AIC, Akaike Information Criterion; aBIC, sample size-adjusted Bayesian Information Criterion; BIC, Bayesian Information Criterion; BLRT, Bootstrap Likelihood Ratio Test; LMR, Lo–Mendell–Rubin Adjusted Likelihood Ratio Test.

The 1-profile model was rejected due to elevated information criterion values. The 3-profile model yielded marginally lower AIC, BIC, and aBIC values than the 2-profile solution and showed acceptable entropy (0.801). However, the LMR test was not significant- (*p* = 0.083), and the smallest class comprised only 4.8% of the sample, falling below the prespecified stability threshold. The 4-profile model produced non-significant LMR and BLRT tests (*p* = 0.124 and 0.118, respectively), entropy below 0.80 (0.763), and two classes with <5% of the sample. In contrast, the 2-profile solution exhibited lower AIC, BIC, and aBIC values than the 1-profile model, excellent entropy (0.872), significant LMR and BLRT tests (both *p* < 0.001), and both classes exceeding the 5% threshold (56.7% and 43.3%).

Accordingly, the 2-profile solution was retained as the optimal model by jointly considering the statistical fit, class size, interpretability, and downstream analytic feasibility. Because the six FCS-CHF dimensions were moderately to highly correlated, these profiles should be interpreted as reflecting graded differences in overall coping levels rather than strongly distinct qualitative types of coping.

### Interpretation of profiles

**Figure 1:**
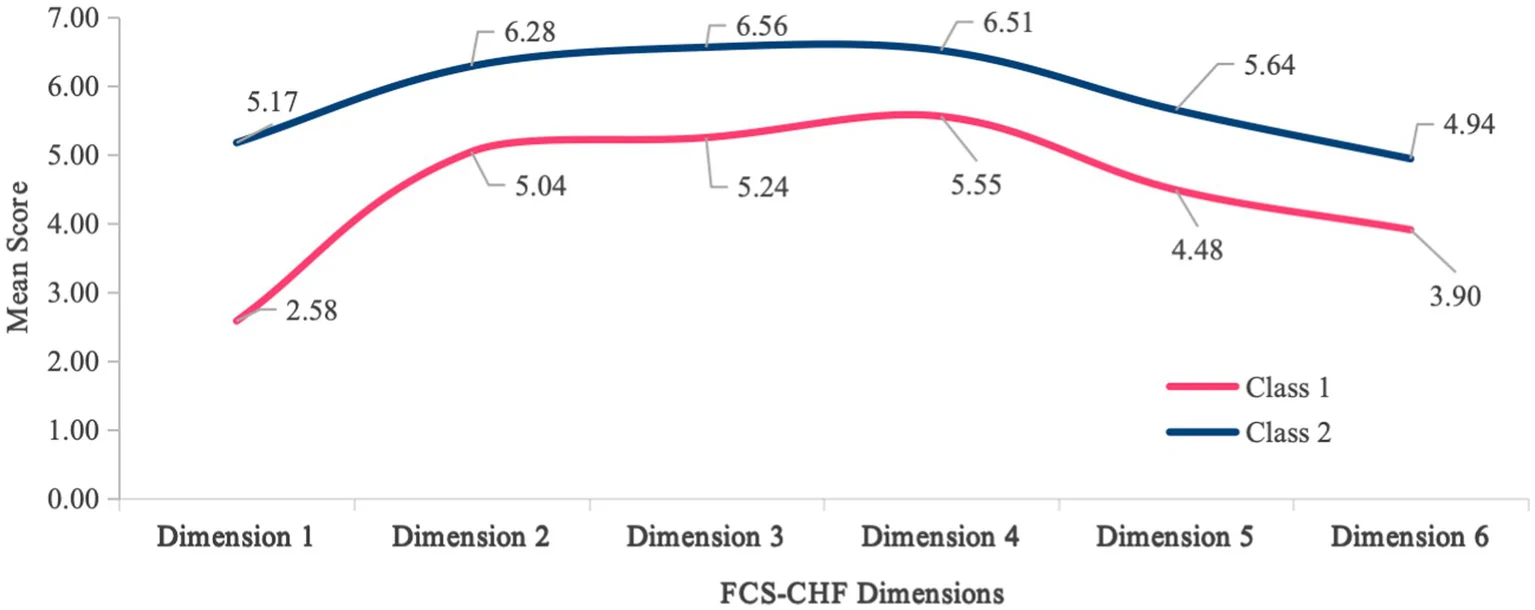
Profiles of family coping across six FCS-CHF dimensions. Class 1 (Depleting Family Coping, *n* = 232, 43.3%) scored consistently lower across all six dimensions than Class 2 (Constructive Family Coping, *n* = 304, 56.7%). Error bars represent 95% confidence intervals. Dimension 1: Strategies for better management of CHF; Dimension 2: Psychological coping; Dimension 3: Substantial support by family members; Dimension 4: Emergency coping; Dimension 5: Overall heart failure awareness; Dimension 6: Patients’ health behavior.

Figure 1 displays the two profiles across the six FCS-CHF dimensions. Profile 1 (Constructive Family Coping, *n* = 304, 56.7%) scored consistently higher than Profile 2 (Depleting Family Coping, *n* = 232, 43.3%) on all six dimensions: strategies for better CHF management, psychological coping, substantial family support, emergency coping, overall heart failure awareness, and patients’ health behaviors.

The mean profile plot shows two largely parallel curves, with Profile 1 maintaining higher scores across all dimensions and Profile 2 consistently showing lower scores. This ordered pattern suggests that the retained solution primarily captures graded differences in overall coping levels rather than qualitatively distinct ones. Given the moderate to high correlations among the six FCS-CHF dimensions, these profiles should be interpreted as reflecting a quantitative continuum of family coping resources rather than strongly differentiated latent types.

Based on the latent profile analysis, Profile 1 was designated as the Constructive Family Coping group, which was characterized by consistently higher coping resources across all measured domains. Profile 2 was designated as the Depleting Family Coping group, reflecting a uniformly lower coping capacity.

### Predictor of latent profile membership

Chi-square tests revealed significant differences between the two profiles in terms of place of residence (χ^2^ = 6.181, *p* = 0.013), educational level (χ^2^ = 7.064, *p* = 0.008), occupation (χ^2^ = 7.823, *p* = 0.020), self-reported health status (χ^2^ = 27.630, *p* < 0.001), family model (χ^2^ = 8.222, *p* = 0.042), family size (χ^2^ = 14.517, *p* < 0.001), and annual household income (χ^2^ = 29.197, *p* < 0.001). T-tests indicated that the two groups differed significantly on all FCS-CHF dimensions and CGFFS scores. The detailed group characteristics are presented in Tables 1, 3.

**Table 3:** Descriptive statistics for the indicator variables that constitute the two profiles.

Variables	Constructive family coping (M ± SD)	Depleting family coping (M ± SD)	*t*	*p*
the Family Coping Scale for Patients with Chronic Heart Failure	140.37 ± 10.49	103.51 ± 13.58	−35.463	<0.001
Strategies for better management of CHF	31.21 ± 5.34	15.52 ± 6.48	−29.931	<0.001
Psychological coping	31.44 ± 3.26	25.25 ± 5.21	−15.895	<0.001
Substantial support by family members	26.27 ± 2.44	21.01 ± 3.81	−18.361	<0.001
Emergency coping	19.61 ± 1.67	16.59 ± 3.18	−13.182	<0.001
Overall heart failure awareness	16.96 ± 2.59	13.47 ± 4.04	−11.497	<0.001
Patients’ health behavior	14.88 ± 4.32	11.68 ± 3.43	−9.558	<0.001
Chinese version of the Chalder Fatigue Scale (CChFS)	5.23 ± 3.64	6.06 ± 3.34	2.687	0.007
Chinese version of General Family Functioning Scale (CGFFS)	3.19 ± 0.35	3.03 ± 0.36	−5.174	<0.001

Binary logistic regression was conducted with coping profile membership as the dependent variable (constructive family coping = 0, depleting family coping = 1). The results are shown in Table 4. All odds ratios (ORs) were interpreted with the constructive family coping group as the reference: OR > 1 indicated higher odds of belonging to the depleting family coping profile, and OR < 1 indicated lower odds.

**Table 4:** Binary logistic regression results predicting profile membership.

Variables	*B*	SE	Wald χ^2^	*p*	OR	95% CI	
						Low	High
*Constant*	3.940	1.008	15.283	<0.001	51.422	—	—
Place of residence (Rural / Urban)	−0.122	0.278	0.195	0.659	0.885	0.513	1.524
Educational level (Primary school or below / Junior high school or above)	−0.118	0.216	0.297	0.586	0.889	0.582	1.357
*Occupation (the farmer or worker as the reference)*			1.004	0.605			
Retired	0.016	0.304	0.003	0.959	1.016	0.560	1.842
Other	−0.277	0.326	0.722	0.396	0.758	0.400	1.436
*Self-rated health status (the very good as the reference)*			10.012	0.018			
Good	−0.672	0.406	2.741	0.098	0.511	0.231	1.131
Fair	0.088	0.386	0.053	0.819	1.093	0.513	2.328
Poor	−0.147	0.466	0.099	0.753	0.864	0.347	2.151
*Family model (the nuclear family as the reference)*			5.447	0.142			
Stem family	0.553	0.280	3.894	0.048	1.739	1.004	3.013
Extended family	0.174	0.349	0.249	0.618	1.190	0.600	2.360
Other	−0.220	0.367	0.360	0.549	0.802	0.391	1.648
*Family Size (the ≤ 2 persons as the reference)*			8.765	0.012			
3–6 persons	−0.852	0.293	8.444	0.004	0.427	0.240	0.758
≥7 persons	−0.566	0.457	1.532	0.216	0.568	0.232	1.391
*Annual Household Income (the ≤ 50,000 CNY as the reference)*			6.957	0.138			
50,001–100,000 CNY	−0.219	0.264	0.686	0.407	0.804	0.479	1.348
100,001–150,000 CNY	−0.070	0.334	0.043	0.835	0.933	0.485	1.796
150,001–200,000 CNY	−0.659	0.390	2.861	0.091	0.517	0.241	1.110
≥210,000 CNY	−0.880	0.392	5.030	0.025	0.415	0.192	0.895
Chinese version of the Chalder Fatigue Scale (CChFS)	0.049	0.027	3.137	0.077	1.050	0.995	1.108
Chinese version of General Family Functioning Scale (CGFFS)	−1.168	0.276	17.857	<0.001	0.311	0.181	0.535

Better family functioning (higher CGFFS scores) was associated with lower odds of depleting family coping (OR = 0.311; 95% CI: 0.181–0.535; *p* < 0.001). Medium family size (3–6 persons), compared with small families (1–2 persons) as the reference, was associated with lower odds of depleting family coping (OR = 0.427, 95% CI: 0.240–0.758; *p* = 0.004). Higher annual household income (≥200,000 CNY), compared with the lowest income category (<30,000 CNY), was associated with lower odds of depleting family coping (OR = 0.415, 95% CI: 0.192–0.895; *p* = 0.025). Conversely, the stem family model, compared to the nuclear family model as the reference, was associated with higher odds of depleting family coping (OR = 1.739, 95% CI: 1.004–3.013; *p* = 0.048).

All ORs describe the statistical associations observed in this cross-sectional sample and do not imply causality. Terms such as “protective” or “risk” have been avoided throughout the text to prevent causal misinterpretations.

## Discussion

This study employed latent profile analysis to identify distinct family coping profiles among patients with CHF and examined their associations with sociodemographic factors, family functioning, and fatigue. The optimal solution comprised two profiles—constructive family coping (56.7%) and depleting family coping (43.3%)—determined using multiple fit indices. Notably, the two profiles exhibited a monotonic pattern: the constructive family coping profile scored uniformly higher across all six FCS-CHF dimensions. This pattern suggests that in this rural, resource-limited population, family coping varies primarily by the degree of coping resources rather than qualitatively distinct coping styles.

Although a monotonic pattern resembles a high–low split of total scale scores, LPA offers three distinct advantages over a simple continuous approach: empirical determination of subgroup number without arbitrary cut-points, probabilistic classification that accounts for measurement uncertainty, and formal testing of whether additional profiles are warranted (in this case, they were not). Thus, the monotonic pattern represents an empirical finding rather than a methodological artifact, indicating that in this specific clinical population, family coping operates along a quantitative continuum (Rolland, 1994; Rolland, 2003).

From a clinical perspective, the two profiles identified groups with divergent intervention needs. Families with constructive family coping profiles may benefit from maintenance strategies that reinforce existing coping strengths. In contrast, those in the depleting family coping profile—nearly half the sample—require comprehensive multicomponent support that addresses multiple coping dimensions simultaneously. This finding aligns with the existing literature, which posits that family support—through informational, emotional, instrumental, and appraisal assistance—serves as a fundamental coping resource that empowers patients to develop effective management strategies (Ryandini, 2020; Conduah et al., 2025; Zhang et al., 2026).

Several sociodemographic factors were associated with profile membership. Medium family size (3–6 persons) was associated with lower odds of Depleting Family Coping than small families (1–2 persons) (OR = 0.427), suggesting that a moderately sized family provides an optimal balance for distributing caregiving responsibilities without the coordination difficulties of very large households (Adamsons et al., 2022; Fang et al., 2024). Higher annual household income (≥200,000 CNY) was associated with lower odds of Depleting Family Coping than the lowest income category (<30,000 CNY) (OR = 0.415), identifying financial vulnerability as a potentially addressable barrier to Constructive Family Coping (Barnett, 2008; Booysen et al., 2021; Neppl et al., 2021; Zietz et al., 2022). Families in the lowest income bracket may face compounding challenges—including the inability to afford medications, transportation, or respite care—that directly constrain coping capacity, consistent with evidence on catastrophic health expenditure and financial toxicity in chronic illness (Awal Chowdhury et al., 2025; Saylor et al., 2023; Yang et al., 2025). In contrast, the stem family model (multigenerational household) was associated with higher odds of Depleting Family Coping than nuclear families (OR = 1.739). A plausible explanation is that multigenerational living may concentrate caregiving responsibilities on fewer members and create intergenerational tensions around treatment decisions, both of which can complicate effective coping (Mitrani et al., 2005; Han et al., 2014; Xiao et al., 2024). Taken together, these findings suggest that clinically meaningful family coping assessments should extend beyond individual-level factors to encompass structural and economic contexts, using these factors as practical screening indicators to identify families who may benefit from targeted, profile-specific interventions.

Better family functioning was strongly associated with the constructive family coping profile (OR = 0.311). This association suggests that families characterized by clearer communication, more effective problem-solving, and healthier role distribution are more likely to be classified into the constructive family coping profile, a finding consistent with family systems theory, which posits that well-functioning families effectively buffer external stressors (Wang et al., 2017; Xiao et al., 2024; Zhang et al., 2026). However, due to its cross-sectional design, the direction of this relationship could not be determined. It is equally plausible that constructive family coping contributes to better perceived family functioning over time, or that the two constructs mutually reinforce one another.

Patient fatigue was not significantly associated with coping profile membership in the fully adjusted model, although a bivariate association was observed. Several *post-hoc* explanations are possible, but none have been directly tested. First, fatigue in CHF may be predominantly determined by physiological factors (e.g., cardiac output, anemia, and systemic inflammation) that overwhelm any psychosocial influence of family coping (Pavlovic et al., 2022; Boulet et al., 2024; Zhang J. et al., 2024). Second, the cross-sectional design may obscure temporal dynamics, such as long-term effects or reverse causality. However, we caution that these interpretations are speculative and hypothesis-generating rather than definitive conclusions. Future research should employ longitudinal designs to examine the specific pathways linking fatigue subtypes and family coping.

This study found that family functioning is associated with family coping among patients with CHF. In the present study, family coping refers specifically to patients’ perceptions of the strategies their families employ to manage CHF-related challenges, whereas family functioning captures the broader quality of family relationships, communication, and role performance (Epstein et al., 1983; Folkman, 2013). Although theoretically distinct, these constructs are not independent. Quantitative studies have demonstrated that they load onto separate factors and exhibit different associations with external criteria (Zhang X. et al., 2024). Qualitative research has further revealed that families distinguish between “getting through daily illness tasks” and “how we generally get along” (Zhang et al., 2026).

According to Rolland’s Family Systems Illness Model (Rolland, 2025), coping operates as a proximal mechanism—the specific strategies and behaviors families enact in direct response to illness-related stressors—whereas family functioning represents a broader, more distal system characteristic that both enables effective coping and may be enhanced by successful coping over time. This distinction has important clinical implications: interventions may target coping directly (e.g., teaching specific CHF management strategies) while also addressing functioning (e.g., improving family communication) to create an enabling context for sustained adaptive coping (Kitko et al., 2020; Rolland, 2025).

In the Chinese cultural context, filial expectations shape coping (e.g., caregiving decisions, emergency preparedness), whereas family harmony beliefs more strongly affect functioning (e.g., communication, conflict resolution) (Xiao et al., 2024; Yu et al., 2025). The traditional belief that “harmony within the family brings prosperity to all endeavors” underscores the role of family functioning in addressing coping and health challenges (Xiao et al., 2024; Zhang X. et al., 2024; Yu et al., 2025). Therefore, the observed association should be interpreted with this reciprocal relationship in mind and with recognition that shared method variance may inflate the association. Future practice should make the assessment and enhancement of family functioning a core intervention strategy, as strengthening communication and collaboration within families can enhance long-term resilience in coping with chronic diseases. Future multi-informant longitudinal designs are needed to disentangle temporal dynamics.

## Limitations

This study had several limitations. *Causal inference*. The cross-sectional design precludes causal inferences. Longitudinal studies with repeated measures are needed to establish temporal precedence and examine how family coping profiles evolve over the disease trajectory.

*Generalizability*. The sample was recruited from a single province (Yunnan) and excluded patients with NYHA class IV, limiting generalizability. The sample was predominantly rural, with a large proportion of individuals reporting low levels of educational attainment. These characteristics reflect the demographic reality of patients with CHF in resource-limited regions—an underserved population frequently underrepresented in research—and thus, provide valuable empirical data from this specific context. Nevertheless, the findings may not be generalizable to urban populations, patients from economically advantaged regions, or those with higher health literacy levels. Future research should replicate these findings in diverse samples, including multiple geographic regions and international settings in China and other countries.

*Profile solution*. The 2-profile solution, while statistically optimal in the present sample, exhibited a monotonic pattern: the Constructive Family Coping profile scored uniformly higher across all six dimensions than the Depleting Family Coping profile, rather than showing a crossover pattern of divergent strengths and weaknesses. This raises the question of whether the LPA solution recapitulates the high–low split of total scale scores, a limitation that the present study acknowledges.

Nevertheless, the monotonic pattern represents an empirical finding rather than a methodological artifact. LPA offers three distinct advantages over a simpler continuous approach: (1) the number of subgroups is determined empirically through fit indices rather than arbitrary cut-points; (2) probabilistic classification accounts for measurement uncertainty, which is clinically relevant for borderline cases; and (3) formal statistical tests confirm that solutions with more than two profiles are not statistically supported. Thus, despite its monotonic nature, the two-profile- solution captures meaningful heterogeneity in family coping resources rather than merely dichotomizing a continuous score. Future research with larger and more socioeconomically diverse samples should examine whether non-monotonic profiles emerge.

### Strengths

Despite these limitations, this study had several strengths that should be noted. To our knowledge, this is the first study to apply latent profile analysis to examine family coping patterns in patients with CHF. The inclusion of a large sample (*N* = 536) from a historically underrepresented region provides novel empirical data on family coping processes in a rural, low-education, and resource-limited context. These findings complement existing research on urban and economically advantaged populations, contributing to a more comprehensive understanding of family adaptation to chronic illness across diverse social and geographic strata.

### Clinical implications

Despite these limitations, the identification of two distinct family coping profiles provides a clinically actionable framework for future research. The routine assessment of family coping using brief instruments could help identify families with depleting family coping profiles who may benefit from comprehensive support programs. In resource-limited settings, such as rural southwestern China, interventions should be designed with the recognition that families with fewer members, lower incomes, and multigenerational structures may face compounding challenges that require integrated, family-centered approaches rather than single-dimension support. Importantly, among patients with NYHA class I–III CHF, the population examined in this study, these findings provide a useful framework for identifying at-risk families. However, these findings may not be generalizable to NYHA class IV patients, in whom family coping demands are likely to be more intense; therefore, dedicated research is needed.

## Conclusion

This study employed latent profile analysis to characterize patient-perceived family coping profiles among patients with chronic heart failure, identifying two distinct subgroups: constructive family coping (56.7%) and depleting family coping (43.3%).

Based on these findings, this study has several implications for clinical practice and future research in rural settings with limited resources. Clinically, routine assessment of family coping using brief instruments could help identify families with depleting coping profiles, who may benefit from targeted family centered support. Healthcare services should consider integrating family resource evaluation, including household size, economic strain, and family functioning, into routine CHF care to develop personalized and integrated intervention strategies.

## Data Availability

The original contributions presented in the study are included in the article/supplementary material, further inquiries can be directed to the corresponding author.
