# Correction: Family coping profiles in patients with chronic heart failure: a latent profile analysis

**DOI:** 10.3389/fpsyg.2026.1951178

**Published:** 2026-09-02

**Authors:** Xiong Zhang, Yimei Zhang, Xu Su, Wei Wei, Yangjuan Bai, Fang Ma

**Affiliations:** 1 Department of Geriatric General Surgery, The First Affiliated Hospital of Kunming Medical University, Kunming, Yunnan, China; 2 Department of Critical Care Medicine, The First Affiliated Hospital of Kunming Medical University, Kunming, Yunnan, China; 3 Department of Psychiatry, The First Affiliated Hospital of Kunming Medical University, Kunming, Yunnan, China; 4 Department of Digestive Surgery, The First Affiliated Hospital of Kunming Medical University, Kunming, Yunnan, China; 5 Department of Cardiology, The First Affiliated Hospital of Kunming Medical University, Kunming, Yunnan, China; 6 Department of Nursing, The First Affiliated Hospital of Kunming Medical University, Kunming, Yunnan, China

**Keywords:** family coping, psychological, chronic heart failure, latent profile analysis, nursing

Affiliation Department of Geriatric General Surgery, The First Affiliated Hospital of Kunming Medical University, Kunming, Yunnan, China was omitted from the paper. This affiliation has now been added for author Xiong Zhang.

Affiliation Department of Critical Care Medicine, The First Affiliated Hospital of Kunming Medical University, Kunming, Yunnan, China was omitted from the paper. This affiliation has now been added for author Yimei Zhang.

Affiliation Department of Psychiatry, The First Affiliated Hospital of Kunming Medical University, Kunming, Yunnan, China was omitted from the paper. This affiliation has now been added for author Xu Su.

Affiliation Department of Digestive Surgery, The First Affiliated Hospital of Kunming Medical University, Kunming, Yunnan, China was omitted from the paper. This affiliation has now been added for author Wei Wei.

Affiliation Department of Cardiology, The First Affiliated Hospital of Kunming Medical University, Kunming, Yunnan, China was omitted from the paper. This affiliation has now been added for author Yangjuan Bai.

Affiliation Department of Nursing, The First Affiliated Hospital of Kunming Medical University, Kunming, Yunnan, China was omitted from the paper. This affiliation has now been added for author Fang Ma.

Affiliation Department of Dry Surgery (Geriatric General Surgery), First Affiliated Hospital of Kunming Medical University, Kunming, China was erroneously included in the paper. This affiliation has now been removed for all author(s).

There was a mistake in Tables 1, 4 as published. The sign of the chi-square value in the tables (χ^2^) was incorrect. The corrected Tables 1, 4 appear below.

**Table 1:** The descriptive statistics for the full sample and each latent profile.

Variables	Categories	Total (*N* = 536)	Constructive family coping (*N* = 304)	Depleting family coping (*N* = 232)	χ^2^	*P*
Sex	Male	311 (58.02)	184 (60.53)	127 (54.74)	1.808	0.179
	Female	225 (41.98)	120 (39.47)	105 (45.26)		
Ethnicity	Han	447 (83.40)	261 (85.86)	186 (80.17)	3.069	0.080
	Minority	89 (16.60)	43 (14.14)	46 (19.83)		
Age	< 65 years	201 (37.50)	117 (38.49)	84 (36.21)	0.292	0.589
	≥65 years	335 (62.5)	187 (61.51)	148 (63.68)		
Place of residence	Rural	249 (46.46)	127 (41.78)	122 (52.59)	6.181	0.013
	Urban	287 (53.54)	177 (58.22)	110 (47.41)		
Living arrangement	Living with spouse only	174 (32.46)	94 (30.92)	80 (34.48)	4.496	0.213
	Living with children/multiple generations only	148 (27.61)	78 (25.66)	70 (30.17)		
	Living with spouse and children/multiple generations	149 (27.80)	95 (31.25)	54 (23.28)		
	Other	65 (12.13)	37 (12.17)	28 (12.07)		
Educational level	Primary school or below	286 (53.36)	147 (48.36)	139 (59.91)	7.064	0.008
	Junior high school or above	250 (46.64)	157 (51.64)	93 (40.09)		
Marital status	Married	393 (73.32)	221 (72.70)	172 (74.14)	5.382	0.068
	Widowed	119 (22.20)	64 (21.05)	55 (23.71)		
	Other	24 (4.48)	19 (6.25)	5 (2.16)		
Number of children	≤ 2	330 (61.57)	184 (60.53)	146 (62.93)	0.322	0.571
	≥3	206 (38.43)	120 (39.47)	86 (37.07)		
Occupation	Farmer or Worker	275 (51.31)	142 (46.71)	133 (57.33)	7.823	0.020
	Retired	179 (33.40)	106 (34.87)	73 (31.47)		
	Other	82 (15.30)	56 (18.42)	26 (11.21)		
Payment method	Employee basic medical insurance	145 (27.05)	87 (28.62)	58 (25.00)	0.879	0.644
	Urban and rural resident basic medical insurance	341 (63.62)	189 (62.17)	152 (65.52)		
	Other	52 (9.70)	28 (9.21)	22 (9.48)		
Self-rated health status	Very good	42 (7.84)	27 (8.88)	15 (6.47)	27.630	< 0.001
	Good	143 (26.68)	105 (34.54)	38 (16.38)		
	Fair	299 (55.78)	142 (46.71)	157 (67.67)		
	Poor	52 (9.70)	30 (9.87)	22 (9.48)		
Family model	Nuclear family	190 (35.45)	100 (32.89)	90 (38.79)	8.222	0.042
	Stem family	201 (37.50)	110 (36.18)	91 (39.22)		
	Extended family	101 (18.84)	70 (23.03)	31 (13.36)		
	Other	44 (8.21)	24 (7.89)	20 (8.62)		
Family size	≤ 2 persons	160 (29.85)	71 (23.36)	89 (38.36)	14.517	< 0.001
	3–6 persons	331 (61.75)	207 (68.09)	124 (53.45)		
	≥7 persons	45 (8.40)	26 (8.55)	19 (8.19)		
Annual household income	≤ 50,000 CNY	210 (39.18)	95 (31.25)	115 (49.57)	29.197	< 0.001
	50,001–100,000 CNY	130 (24.25)	73 (24.01)	57 (24.57)		
	100,001–150,000 CNY	71 (13.25)	42 (13.82)	29 (12.50)		
	150,001–200,000 CNY	58 (10.82)	42 (13.82)	16 (6.90)		
	≥210,000 CNY	67 (12.50)	52 (17.11)	15 (6.47)		

The original version of this article has been updated.

**Table 4:** Binary logistic regression results predicting profile membership.

Variables	*B*	SE	Wald χ^2^	*P*	OR	95% CI	
						Low	High
Constant	3.940	1.008	15.283	< 0.001	51.422	–	–
Place of residence (Rural/Urban)	−0.122	0.278	0.195	0.659	0.885	0.513	1.524
Educational level (Primary school or below/Junior high school or above)	−0.118	0.216	0.297	0.586	0.889	0.582	1.357
Occupation (the farmer or worker as the reference)			1.004	0.605			
Retired	0.016	0.304	0.003	0.959	1.016	0.560	1.842
Other	−0.277	0.326	0.722	0.396	0.758	0.400	1.436
Self-rated health status (the very good as the reference)			10.012	0.018			
Good	−0.672	0.406	2.741	0.098	0.511	0.231	1.131
Fair	0.088	0.386	0.053	0.819	1.093	0.513	2.328
Poor	−0.147	0.466	0.099	0.753	0.864	0.347	2.151
Family model (the nuclear family as the reference)			5.447	0.142			
Stem family	0.553	0.280	3.894	0.048	1.739	1.004	3.013
Extended family	0.174	0.349	0.249	0.618	1.190	0.600	2.360
Other	−0.220	0.367	0.360	0.549	0.802	0.391	1.648
Family size (the ≤ 2 persons as the reference)			8.765	0.012			
3–6 persons	−0.852	0.293	8.444	0.004	0.427	0.240	0.758
≥7 persons	−0.566	0.457	1.532	0.216	0.568	0.232	1.391
Annual household income (the ≤ 50,000 CNY as the reference)			6.957	0.138			
50,001–100,000 CNY	−0.219	0.264	0.686	0.407	0.804	0.479	1.348
100,001–150,000 CNY	−0.070	0.334	0.043	0.835	0.933	0.485	1.796
150,001–200,000 CNY	−0.659	0.390	2.861	0.091	0.517	0.241	1.110
≥210,000 CNY	−0.880	0.392	5.030	0.025	0.415	0.192	0.895
Chinese version of the Chalder Fatigue Scale (CChFS)	0.049	0.027	3.137	0.077	1.050	0.995	1.108
Chinese version of General Family Functioning Scale (CGFFS)	−1.168	0.276	17.857	< 0.001	0.311	0.181	0.535

